# Current Status and Future Perspectives of Artificial Intelligence in Magnetic Resonance Breast Imaging

**DOI:** 10.1155/2020/6805710

**Published:** 2020-08-28

**Authors:** Anke Meyer-Bäse, Lia Morra, Uwe Meyer-Bäse, Katja Pinker

**Affiliations:** ^1^Department of Scientific Computing, Florida State University, Tallahassee, Florida 32310-4120, USA; ^2^Dipartimento di Automatica e Informatica, Politecnico di Torino, Torino, Italy; ^3^Department of Electrical and Computer Engineering, Florida A&M University and Florida State University, Tallahassee, Florida 32310-4120, USA; ^4^Department of Biomedical Imaging and Image-Guided Therapy, Division of Molecular and Gender Imaging, Medical University of Vienna, Vienna, Austria; ^5^Department of Radiology, Memorial Sloan-Kettering Cancer Center, New York, New York 10065, USA

## Abstract

Recent advances in artificial intelligence (AI) and deep learning (DL) have impacted many scientific fields including biomedical maging. Magnetic resonance imaging (MRI) is a well-established method in breast imaging with several indications including screening, staging, and therapy monitoring. The rapid development and subsequent implementation of AI into clinical breast MRI has the potential to affect clinical decision-making, guide treatment selection, and improve patient outcomes. The goal of this review is to provide a comprehensive picture of the current status and future perspectives of AI in breast MRI. We will review DL applications and compare them to standard data-driven techniques. We will emphasize the important aspect of developing quantitative imaging biomarkers for precision medicine and the potential of breast MRI and DL in this context. Finally, we will discuss future challenges of DL applications for breast MRI and an AI-augmented clinical decision strategy.

## 1. Introduction

Magnetic resonance imaging (MRI), in particular dynamic contrast-enhanced MRI (DCE-MRI), is a non-invasive, well-established breast imaging modality with several indications in oncology including screening of high-risk women, preoperative staging, and therapy monitoring [1].

DCE-MRI interpretation is complex and time-consuming, involving the analysis of hundreds of images. The time-signal intensity curves of multiple postcontrast sequences reflect changes induced by uptake of contrast agent over time and allow the extraction of both spatial and temporal patterns, reflective of both tumor morphology and metabolism. The clinician is thus faced with an increasingly large amount of data per patient to determine a diagnosis. The heterogeneous, complex, and multidimensional data stemming from breast imaging, further integrated by sources of omics data (e.g., genomics), makes it difficult to decipher the clinical meaning.

The US National Research Council recently proposed the development of a new taxonomy for human diseases that integrate the connections between different types of data (clinical, molecular, imaging, genomic, and phenotype) to produce a knowledge network [2]. It is obvious that the effective diagnosis and treatment of an individual patient requires the integration of multiple information sources derived from a large number of patients. Thus, machine learning [3], considered as a subset of AI, has been considered to improve and streamline this process, determining relevant patterns in these data and consequently supporting clinical decision-making [4].

The following are particularly pertinent to radiology and breast imaging: (1) Can imaging capture clinically relevant differences and tumor heterogeneity? (2) Can imaging serve as virtual digital biopsy? (3) Is there a correlation between imaging and genomic features? (4) Can imaging together with genomics improve treatment predictions? (5) Can therapy be decided based on radiogenomics?

In this context, there are an increasing number of clinical and biological features extracted from multiparametric breast imaging techniques that can potentially shed light into these important questions. Imaging data collected during routine clinical examination are an important resource for medical and scientific discovery and for better understanding breast cancer phenotypes. The conversion of these multiparametric images into mineable data has set the framework for a new and exciting translational discipline called “radiomics” [5, 6].

Recent advances in artificial intelligence (AI) have impacted many subspecialties within the field of biomedical imaging. In breast imaging, AI is becoming a key component of many applications, including breast cancer diagnosis, monitoring neoadjuvant therapy, and predicting therapy outcomes.

AI has been around for over sixty years. The term AI has been used lately interchangeably with “pattern recognition” and “deep learning” in the literature, but their meanings are quite different. Indeed, in this paper, we distinguish two classes of pattern recognition algorithms: (a) conventional machine learning (ML) algorithms based on predefined engineered (or hand-crafted) features and (b) deep learning (DL) algorithms. This distinction is adopted by several authors in the biomedical field [7, 8].

The success of DL is based on its ability to automatically learn from data representations with multiple levels of abstractions [9]. This is achieved by composing deep neural networks with multiple processing layers that transform the images into feature vectors (representations), which are then used to discriminate disease patterns, perform segmentation, or other tasks. While DL has become the state-of-the-art approach in computer vision, essentially replacing conventional machine learning for most applications, it is being gradually applied in breast MRI from anatomical segmentation to disease classification. DL analysis of breast MRI is considerably similar to that of advanced computer vision techniques.

In biomedical imaging, conventional ML approaches are still widely applied. Their renaissance stems in particular from the increasing interest in radiomics. In this discipline, “engineered” features describing the radiologic aspects of a tumor such as shape, intensity, and texture are extracted from regions of interest, usually segmented by an expert. Indeed, a recent review suggests that roughly 75% of radiomics studies still rely on hand-crafted features [10]. However, such features are not necessarily optimal in terms of quantification and generalization for a discrimination task. AI and deep learning (DL) have the potential to overcome these challenges and can determine feature representations directly from the images without relying on a time-consuming manual segmentation step.

We believe that the combination of large datasets with DL-powered analysis has the potential to support and improve clinical decision-making in the near future. The ongoing realization of precision medicine is one of the driving forces for implementing AI techniques in breast cancer research. The goal of this narrative review is to analyze how DL is being applied to breast MRI in order to highlight potential benefits, as well as challenges and direction for future applications. We start by providing an overview of fundamental techniques in AI, highlighting differences between conventional ML and DL, and conclude by providing a future perspective on how AI, and DL in particular, will be leveraged for breast MRI in the future.

## 2. Introduction to Data-Driven Approaches in Breast MRI

Data-driven approaches are based on collecting medical imaging data, extracting meaningful features, and learning to classify patterns according to a specific clinical task, e.g., to determine if the specimen is normal or malignant. They are classified into two broad categories: supervised and unsupervised.

Supervised learning requires a class label for training purposes. The training process updates the weights of the trained model by optimizing the difference or error between the computed output and the desired output, given by the correct class label. After training is completed, an unknown pattern can be classified according to the learned weights.

On the contrary, when class labels are missing, we can resort to an unsupervised learning approach. In this case, the training process searches for similarities within the input data, categorizing them into groups or clusters. Similarity can be determined based on a measure of distance, e.g., correlation or Euclidean distance. In the testing phase, an unknown pattern is assigned to the group or cluster to which it is most similar. Unsupervised learning algorithms were often used for lesion segmentation and other image processing tasks, whereas supervised learning is most often used to build predictive models, e.g., to discriminate malignant from benign cases [11].  Table 1 gives an overview of the main data-driven techniques used in breast MRI.

### 2.1. Brief Overview of Traditional Neural Networks and Deep Neural Networks

Artificial neural networks (ANNs) represent simple computational models that mimic information processing in the brain [44, 45]. The ANNs have several processing layers each having a predefined number of neurons. The neurons are connected to each other via weights or synapses. The first layer is the input layer that mimics the neurobiological sensory input information. The following layers process the input information. The output layer is the decision layer regarding the class membership of the unknown input pattern. The number of neurons in the input layer is equal to the number of features describing the unknown input pattern, while the number of neurons in the output layer is equal to the number of different classes/categories to be learned. The number of hidden layers and neurons is problem-oriented. In most applications, neurons and layers are added gradually if needed to improve the overall learning. This type of feedforward ANN architecture processes the patterns from bottom up and saves the information about the learned patterns in the weights. The mutual interconnections are adapted during the learning process to reflect the variations in the input data. ANNs are excellent candidates for processing noisy, inconsistent, or probabilistic information [46].

Multilayer perceptrons (MLPs) represent the first popular type of neural network. They are feedforward ANNs with a prespecified architecture regarding the number of neurons and layers. The weights or interconnections between the neurons are adapted during the learning process. Every single pattern is processed in a forward direction and traverses every single layer.

Like MLPs, deep neural networks have many hierarchical layers and process information progressively from the input to the output layer. DL extracts pattern from high-dimensional image data and uses them as discriminative features, while ML uses hand-crafted features. Recently proposed DL models have multiple layers of nonlinear information processing, feature extraction, and transformation and can be applied for pattern analysis and classification.

Deep learning techniques have emerged as a novel and powerful modality to detect objects in images [9] and are therefore very appealing for processing of biomedical images. They are characterized by their depth, i.e., the number of hidden layers between the input and output layers, which can range between 6 and 7 layers up to the hundreds of most recent applications [47, 48].

The most common DL architecture in image analysis is the convolutional neural network (CNN) [8, 9, 47]. The main component of CNNs is the convolutional layer, which is composed by a series of trainable convolution-based filters, and transforms the input into a feature map. The use of convolutional filters reduces the number of parameters compared to traditional MLPs since weights are shared across the entire input space and supports hierarchical representations by stacking convolutional layers on top of each other. It was empirically observed that different layers serve different purposes. The first layer learns the presence or absence of edges at particular orientations, intensity or color patches, and other low-level image features. The second layer finds motifs by identifying particular arrangements of edges independent of local edge variations. The third layer combines these motifs together into larger combinations corresponding to parts of known objects. Subsequent layers continue the assembling process and detect objects as the result of these combinations. The convolutional filters are trained along with the final classifier in an end-to-end procedure unifying in the same learning framework, i.e., both feature extraction and classifier training. Several variants of CNNs have been proposed in order to perform different visual tasks including classifications, object detection, and segmentation. A detailed overview of base and more advanced neural network architectures is provided in recent publications [8, 49].

### 2.2. Conventional Machine Learning versus Deep Learning

Machine learning techniques have become more sophisticated over the years and have improved in their performance. However, especially, the traditional neural networks have seen a renaissance in the past few years due to an increase in computation power and big data. DL is the result of these two developments.

Traditional neural networks (as well any other conventional ML technique) cannot directly process image data. Thus, a computer-aided diagnostic system requires careful engineering and expert knowledge to design a feature extractor that transforms the pixel values of the image into a suitable feature vector. This feature extraction process usually requires many steps, including normalization, segmentation of the lesion boundary, and then feature extraction [15]. This feature vector then serves as the input of a classifier that detects important clinical patterns of the image. Likewise, many ad hoc algorithms were developed for breast and lesion segmentation [50, 51].

**Figure 1:**
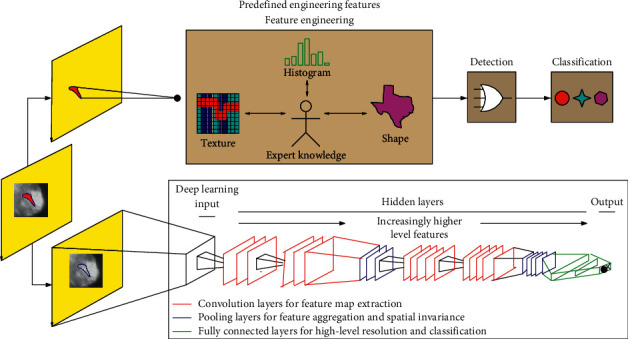
Differences between conventional and deep learning in breast MRI for the lesion discrimination task. The upper part of the image represents the traditional radiomic-based processing. Features such as texture, shape, and histogram are fused to describe the tumor. These engineered features are defined based on expert knowledge. They are extracted from an accurate segmentation which may be performed automatically or, more often, in a semiautomatic fashion by an expert radiologist. The lower part shows the DL-based processing. Several deeper layer features from low level (edges) to high level (objects) are automatically learned by the network. This approach does not require an explicit segmentation step and can be directly applied to the raw images, trained only from lesion-level class labels.

Deep learning belongs to the group of representation learning techniques, which learn directly the optimal representation by optimizing a loss function, e.g., a classification loss. The most important aspect of DL, which significantly departs from conventional ML techniques and traditional neural networks, is the fact that these layers of low- to high-level features are not designed by human engineers but are learned based on representation learning.  Figure 1 exemplifies the differences between conventional and deep learning in breast lesion classification.

Deep learning faces two major challenges in medical imaging: (1) effectively training deep learning neural networks requires very large annotated datasets, and (2) the joint analysis of multimodal images requires high-level features that extract the global and local information hidden in these images.

Training a large neural network from “scratch” (i.e., from random initialization) requires thousands or millions of data points. Despite recent progresses, collecting large-scale dataset is still difficult in the medical domain [52]. A possible compensating strategy is to transfer knowledge from domains where data are abundant. The most standard procedure for transfer learning exploits existing network architecture pretrained on large datasets like ImageNet [53]. The CNN can be used as an off-the-shelf feature extractor, in which case only the final classifier is trained; alternatively, the network can be fully or partially fine-tuned with a limited amount of medical images [54].

The second challenge is related to the nature of breast MRI imaging, which provides complex three-dimensional anatomical and functional information. In DCE-MRI, multiple scans are acquired at different time intervals before and after intravenous contrast injection. In the multiparametric MRI setting, this is pushed even further by combining conventional T1-weighted (T1W) and T2-weighted (T2W) images, diffusion-weighted imaging (DWI), and DCE-MRI sequences, where each sequence provides a distinct contrast yielding a unique signature for each tissue type [55, 56]. In conventional ML, ad hoc features were defined to take into account spatiotemporal image variations, e.g., to properly define tumor kinetics, often extracted after a preliminary coregistration step which aligns all imaging volumes to reduce motion artifacts [15, 57]. CNNs were initially proposed to deal with two-dimensional, low-resolution, RGB images and therefore need to be adapted in order to effectively process multiparametric inputs and encode both volumetric (spatial) and temporal changes [56]. When transfer learning from ImageNet, researchers have proposed creative solutions to exploit pretrained CNNs by mapping different timepoints or anatomical planes to different input channels [32, 58–61]. In general, DL offers unprecedented opportunities to extract high-level features from multiple low-level images and may also alleviate the need for an intermediate registration step [62].

## 3. Materials and Methods

The primary goal of this narrative review was to identify the most important applications and current research trends in DL applied to breast MRI. A thorough search was conducted in the key databases in the biomedical and engineering domains, i.e., Springer Link, Web of Science, IEEE Xplore, PubMed, and Google Scholar, using the search keywords “breast cancer,” “MR imaging,” and “deep learning.” Additional studies were retrieved by cross-checking reference lists from extracted articles or based on the authors' experience. Only original research articles published as full text and in English were considered. Given that the introduction of deep learning in medical imaging is relatively new [49] and the rapid pace of technological evolution, only articles published after 2016 were included in the search. Two authors reviewed the titles and abstracts for relevance, e.g., to exclude papers that pertained to other types of cancers or anatomical districts, other imaging modalities, or not based on deep learning. The following studies were excluded: reviews, systematic reviews, editorials and letters, opinion papers, and articles that did not include a description of the methodology. We included conference proceedings and preprints that are widely used by the engineering and computer science communities.

The primary aim was to categorize the studies according to the following research questions: (1) what are the main applications of DL in breast MRI? (2) What are the DL architectures currently applied in breast MRI? (3) What are the evaluation criteria used for their assessment? (4) What are the datasets used? (5) What are their performances? Therefore, a systematic approach to data extraction was followed to produce a descriptive summary of study characteristic. Each study was categorized according to the main task that the methodology was designed to solve and assigned to one of the following categories: segmentation, lesion detection, lesion classification, radiomics, predictive modeling, and others [11]. We further analyzed the most important applications by extracting the following information: description of the DL technique, dataset characteristics (size and type of sequences), and performance. When multiple articles were published on the same technique or dataset, the most recent or complete work was included in the systematic review.

## 4. Perspectives of AI and Deep Learning in Breast MRI

**Figure 2:**
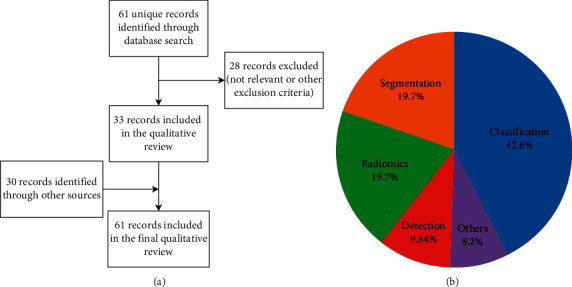
Flowchart shows selection of studies for inclusion in the narrative review (a); selected studies are further characterized according to the main focus (b).

Overall, 61 studies were considered in this systematic review, as detailed in Figure 2.

The majority of studies falls within the broad scope of computer-aided detection/diagnosis. Twelve studies (20%) focus on segmentation of either the breast region (5 studies) or the lesion boundaries (7 studies), which is a key preprocessing step for many subsequent applications.

Only 6 (10%) studies focus on automatic lesion detection or Computer-Aided Detection (CADe) applications, whereas 26 (42%) focus on classification of benign vs. malignant lesions or Computer-Aided Diagnosis (CADx). The very high sensitivity of breast MRI, in addition to its primarily diagnostic role, has traditionally shifted the interest of researcher towards CADx applications.

CADe applications are designed to automatically detect and localise breast lesions, usually to serve as a second-opinion, reduce the risk of false negatives, and streamline the reading process. The output may be a bounding box or other marker, which indicates the lesion [63] or, more commonly in breast MRI, a pixel-wise segmentation mask [64, 65]. In breast DCE-MRI, sensitivity and prevalence are usually very high, but the reading process is complex and time-consuming: for this reason, CADe developers have been traditionally focused on reducing reading time and provide more reproducible results than manual segmentation [51].

CADx systems may start from the output of a CADe system or, more frequently, from an input ROI, usually a bounding box, manually delineated by the radiologist. A segmentation algorithm may be used to locate the lesion boundary for volumetric analysis or feature extraction. Lesion detection, segmentation, and classification are often tackled as separated, consecutive processing steps, and hence, most papers focus on either one of these steps. In the remainder of this chapter, we will follow this distinction to focus on the unique characteristics of each task. However, the reader must bear in mind that, in DL, it is usually beneficial, in terms of performance and computing time, to combine multiple tasks in a single architecture, a technique usually denoted to as multitask learning. For this reason, several authors are increasingly tackling multiple tasks, e.g., lesion segmentation and classification, simultaneously [64].

Finally, an additional 12 studies (20%) focus on extraction of biomarkers or predictive models, in particular for the prediction of response to neoadjuvant chemotherapy (9 studies). The remaining five studies (8%) include additional applications such as the estimation of breast density [34] or issues related to normalization and preprocessing of MRI data [62, 66–68]. Our findings are consistent with previous reviews and with clinical indications for breast MRI, which include screening of high-risk women, characterization of equivocal findings at conventional imaging, presurgical staging, therapy response monitoring, and searching for occult primary breast cancer [1, 11].

Sections 4.1–4.4 review segmentation, detection, classification, and biomarker applications, respectively.

### 4.1. Segmentation

Segmentation is a key preprocessing step for both CADx and radiomic applications.  Table 2 shows a summary of papers describing segmentation applications in DL.

Some studies have focused on the identification of the breast region, which consists in the identification of the breast-air and breast-pectoral muscle edges, usually with the goal of removing unwanted pixels from further computation [61, 69, 71, 73, 74]. The main challenge is detecting the ill-defined boundary between the breast and the pectoral muscle, which is further complicated by the presence of the heart and wide intersubject variability.

Other authors have focused on the segmentation of lesion boundaries [60, 70, 72, 74–77]. The uneven class distribution between malignant and benign lesions, the presence of small lesions in large image matrices, and the presence of other neighboring anatomical structures such as vessels and breast parenchyma represent the main challenges to accurate lesion segmentation.

The primary evaluation method for biomedical image segmentation is the Dice coefficient [78]. The Dice coefficient is a measure of spatial overlap ranging from 0, indicating no spatial overlap between two sets of binary segmentation results, to 1, indicating complete overlap. It is computed as follows:

(1) $$\begin{array}{c} \text{Dice}\left(S,\text{GT}\right)=2\frac{\left|S\cap \text{GT}\right|}{\left|S\right|+\left|\text{GT}\right|}, \end{array}$$

where |*S*∩GT| is the area of the overlap between the segmentation *S* and the ground truth GT and |*S*| and |GT| are the areas of the segmentation and ground truth, respectively. Since the task of segmentation can be represented as a voxel-by-voxel classification, where each voxel is assigned to a distinct class, it is also common to report the by-voxel accuracy (ACC), sensitivity (Sn), and specificity (Sp).

**Figure 3:**
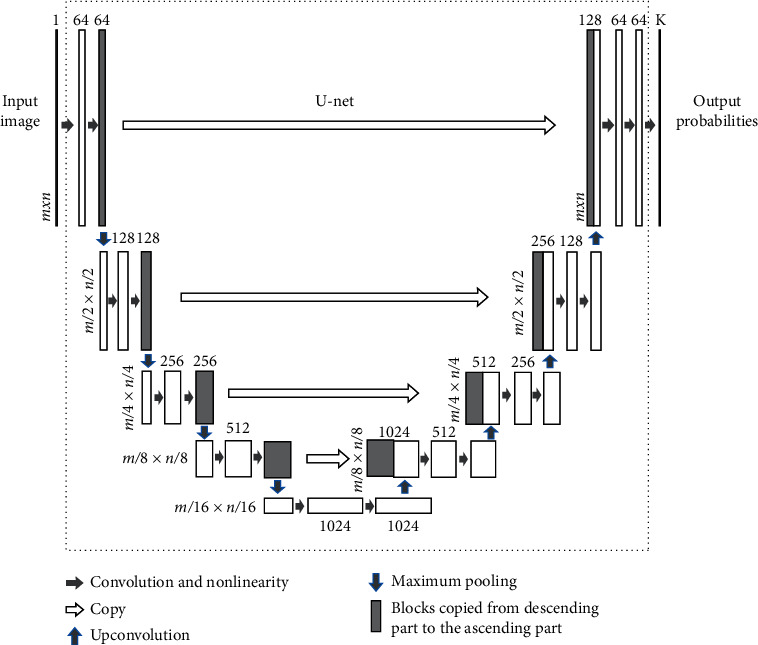
Deep learning network with U-net architecture. Reprinted with permission from [34].

U-net [79] represents the state-of-the-art of segmentation in biomedical image processing and is indeed the most widely used technique for both lesion and breast segmentation [60, 69, 71, 72]. The U-net architecture (shown in Figure 3) builds upon the fully convolutional network and is composed of two sections: a descending part, which compresses the input in a semantically rich latent space to capture context, and an ascending part, which outputs a segmentation map with K channels, one for each type of tissue. The U-net architecture is symmetric and introduces skip connections between the downsampling and upsampling paths, which provide both local and global information to the upsampling convolutions and allow precise localization of each pixel. Despite the 3D nature of breast MRI, almost all available techniques apply a 2D U-net to each slice and then collate the results in a 3D volume [60, 61, 69–71, 73]. This allows a substantial saving in model parameters over 3D convolutions; experimentally, both approaches were found to have comparable results [66].

U-net has shown superior performance to other pixel-based, atlas-based, and geometrical-based approaches. Within the field of breast MRI, a head-to-head comparison is provided by Piantadosi et al. [61], who reported a Dice coefficient between 0.9 and 0.96 for deep learning-based approaches, compared to 0.6–0.63 (pixel-based), 0.69–0.92 (geometrical), and 0.69 (atlas-based) for non-deep learning approaches.

In the case of breast segmentation, it is normally sufficient to use the precontrast scan, whereas for enhancing lesions, a combination of pre- and postcontrast scans are needed to detect contrast agent uptake. For instance, Piantadosi et al. used three well-defined temporal acquisitions (precontrast, 2 minutes and 6 minutes after contrast agent injection, also known as the 3TP method) as three separate inputs to a single network [60]. Other authors have directly encoded spatiotemporal information using a combination of recurrent and convolutional neural networks [80].

#### 4.1.1. Segmentation of Fibroglandular Tissue

**Figure 4:**
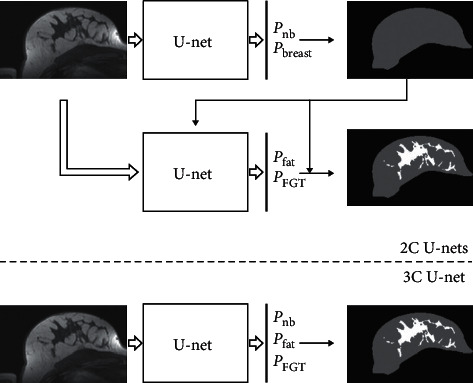
Two different approaches for applying U-net to breast and fibroglandular tissue (FGT) segmentation. The upper figure shows 2C U-nets, where two consecutive U-nets are used. The figure below illustrates the other approach, a single U-net with 3-class outputs. *P*_nb_, *P*_breast_, *P*_fat_, and *P*_FGT_ denote the probability values of voxels to belong to nonbreast, breast, fat, and FGT, respectively. Reprinted with permission from [34].

The breast is composed of fatty and fibroglandular tissue (FGT). Breast density, defined as the percentage of FGT within the breast, is an important aspect of breast cancer diagnosis, as dense breasts are associated with an increased risk of breast cancer and reduced mammography sensitivity [81]. Given the high interrater variability associated with visual assessment [81], automatic breast density estimation has been widely investigated, most commonly based on mammography [82] and, to a lesser extent, on MRI [34, 83, 84]. A possible way to estimate breast density is to classify each voxel as either fat or FGT and thus estimate the percentage of volume occupied by the latter. In this regard, an interesting application of U-net for the segmentation of FGT is presented in [34]. Two different approaches are compared: (1) breast and FGT segmentation performed in two consecutive steps using 2 separate U-nets (2C U-nets) and (2) breast and FGT segmentation performed in a single step using 3-class U-net (3C U-net), as shown in Figure 4. The average Dice values for FGT segmentation obtained from 3C U-net, 2C U-nets, and atlas-based methods were 85.0, 81.1, and 67.1, respectively, thus indicating that the 3C U-net is a more reliable approach for breast density estimation. The authors observe that both U-net-based methods were minimally affected by intensity inhomogeneities typical of MRI even though no bias-field correction was applied as a preprocessing step [85]; this suggests that a deep neural network is able to learn and compensate for the bias field in a given training set [34].

### 4.2. Detection of Breast Lesions

Table 3 shows a summary of papers describing lesion detection applications in DL. While lesion segmentation algorithms, illustrated in Section 4.1, usually operate from a manually defined input ROI, CADe systems operate on the entire volume, with the goal of detecting lesions accurately, i.e., with high sensitivity, low false-positive rate, and good segmentation quality. The output may a bounding box or other marker which indicates the lesion [63] or, more commonly in breast MRI, a pixel-wise segmentation mask [64, 65]. Detection and lesion segmentation may be tackled by a single network or by dedicated submodules.

Evaluation of CADe systems is usually performed by free-response receiver operative curve (FROC) analysis [89]. It is a variant of the receiver operating curve (ROC) paradigm where the number of detections for an image is not constrained, as CADe systems may generate an arbitrary number of lesion candidates. Each lesion candidate is assigned a score, and candidates with score higher than a given threshold (or operating point) are shown to the radiologist.

In particular, the FROC curve plots the fraction of correctly localized lesions as a function of the average number of false positives (FPs) per image, where each point in the curve corresponds to a different threshold. The FROC curve is not bounded; hence, a convenient summary measure like the area under the ROC curve is not readily available. Starting from FROC analysis, the authors may select an optimal operating point at which sensitivity and FPs/image are reported: the choice of the operating point depends on the desired balance between sensitivity and specificity, but it is also possible to select multiple operating points, e.g., corresponding to high-sensitivity or high-specificity settings. For instance, Maicas et al. achieved a sensitivity of 80% at 8 FPs per image using a model agnostic saliency model [86] and 80% sensitivity at 3.2 FPs per image using a method based on deep reinforcement learning [63]. Other authors have selected a computation performance metric (CPM), where sensitivity values at 1/8, 1/4, 1/2, 1, 2, 4, and 8 false positives per scan were averaged [64].

As in the case of lesion segmentation, fully convolutional networks and variants like the U-net architecture are a common choice for lesion detection [64, 87]. Other authors leverage on classification networks that are applied on image patches in a sliding window fashion [65]. Both approaches output a binary segmentation map. Very different implementation choices are available within this same architecture, based on how to exploit the 4D data provided by DCE-MRI. In the case of patch-based classification, sometimes, the ROC curve is used to evaluate how well the network can discriminate lesions from the background; however, this performance metrics is less common as it refers to an intermediate output of the CAD system, and as such, it is not directly interpretable by the end user.

Detection of enhancing lesions requires the processing of postcontrast frames: Herent et al. [65] relied on a single postcontrast fat-suppressed sequence, whereas other authors have used the subtraction volume obtained from precontrast and the first postcontrast volumes, where the lesion is most prominent [64]. The additional T1-weighted (T1W) scans obtained after the first postcontrast MRI are used for evaluating contrast enhancement dynamics of a lesion in the late phase, which provides adjunct information for distinguishing the benign structures from the malignant ones [64]. Here, a modular approach may be useful to reduce the computational time associated with the initial detection step, reserving late frames or multiparametric imaging for targeted classification analysis on the selected ROIs.

An important contribution to breast cancer detection is presented in [64]. The system was based on three-dimensional (3D) morphological information from the candidate locations. Symmetry information arising from the enhancement differences of the two breasts is exploited by implementing a multistream CNN, which simultaneously processes and combines features from the target ROI and the contralateral breast. In a head-to-head comparison, the proposed system achieves a higher average sensitivity (0.6429 ± 0.05387) compared to a previous CADe system (0.5325 ± 0.0547) based on conventional image processing and ML techniques.

There are however other approaches in literature. For instance, Lu et al. [87] took advantage of different image modes from breast MRIs (T1W, T2W, and DWI), building a multistream CNN backbone with shared weights in which features are extracted from each modality, concatenated, and finally input to a classification model. A radically different approach is taken in consideration by Maicas et al. [63], who propose a deep reinforcement learning for accurate lesion detection. In this framework, a network is used to modify (translate or scale) a bounding box proposal until the lesion is found.

### 4.3. Classification of Breast Lesions

Lesion classification according to their histological type (benign vs. malignant) accounts for almost half the research reviewed.  Table 4 shows a summary of the most representative papers.

The vast majority of implementations leverages a classification network that takes as input a region of interest (ROI) containing the lesion and outputs a classification score. Usually, a precise segmentation is not performed as it is not needed for DL-based methods.

One of the first CNN implementations can be traced back to Antropova et al. [99], who combined off-the-shelf pretrained CNN with SVM. While the architectures vary, following the DL evolution towards deeper and deeper architectures, leveraging on a pretrained on ImageNet has remained very popular in the literature, although more recent works have shown that fine tuning all layers towards the task of breast MRI classification is needed to achieve high performance [32, 58, 93, 94, 96, 97, 100–102].

**Figure 5:**
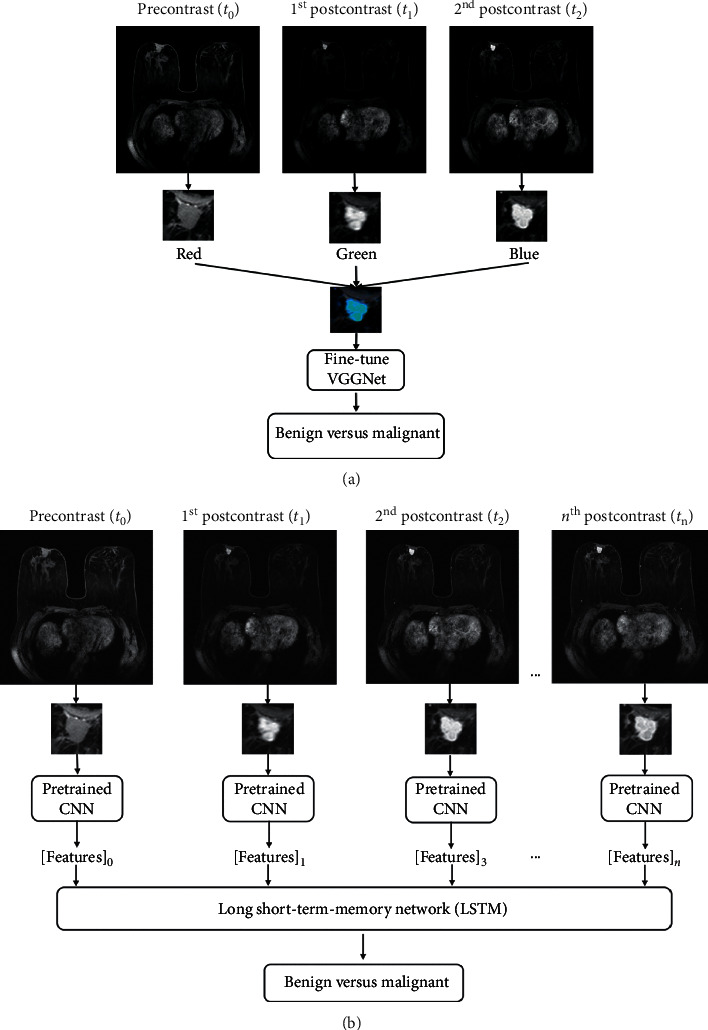
Two-step transfer learning approach for leveraging temporal information in pretrained CNN. In the first approach (a), the CNN is fine-tuned on pseudocolor ROIs, formed by the precontrast and first and second postcontrast frames, mimicking the three channels of an RGB images. In the second step (b), image features extracted from the trained CNN at each DCE timepoint are used to train an LSTM network, which learns to distinguish contrast enhancement patterns. Reprinted from [58].

As for the previous tasks, different variations are available depending on how information is combined as input to the pretrained CNN. Since natural images are RGB (three channels), whereas MRI is grayscale (single channel), this gives the option to input different pre- and postcontrast frames to different channels: to this aim, it is possible to adopt the 3TP method [97] or use the precontrast, first postcontrast, and second postcontrast frames, as shown in Figure 5 [100]. Fewer authors have evaluated multiple combination of sequences or multimodal including DCE-MRI, T2-weighted MR, and DWI [91, 93, 94]. Our findings are consistent with previous reviews which included also conventional ML methods [11].

One of the most challenging aspects of designing deep neural networks for breast MRI is integrating both temporal and spatial aspects in feature extraction, especially when constrained by the available networks designed for 2D images. In this direction, Antropova et al. [32] exploited maximum intensity projection (MIP) in order to integrate spatial information and used subtraction images to compare pre- and postcontrast frames, effectively reducing the 4D volume to a 2D image, while retaining information about enhancement changes throughout the whole lesion volume. Hu et al. [101] introduced a pooling layer to reduce the images at the feature level, instead of the image level, as in the MIP case.

Recurrent neural networks, such as long short-term memory (LSTM), were also applied to the task of lesion classification [37, 58, 92]. Morphological features are captured by a CNN on each ROI, and then, the extracted features at different time points are used to train a LSTM network to predict the outcome based on the full DCE-MRI sequence. An example of recurrent neural network is given in Figure 5.

Fewer authors have proposed ad hoc CNN architectures, leveraging directly the 4D nature of DCE-MRI, for instance, by exploiting 3D convolutional layers [35, 95, 98, 103] and by extracting features at multiple scales [95]. These approaches are particularly interesting as they allow to capture the unique properties of DCE-MRI datasets. At the same time, it becomes necessary to train the network from scratch, and this requires relatively large-scale datasets to achieve competitive performance [98].

Comparison of deep learning vs. hand engineered features was performed by several authors [90, 94, 100, 104]. Antropova et al. [100] found that a CNN-based classifier slightly outperformed a conventional CADx design (AUC = 0.87 vs. 0.86), and a combination of both approaches performed best (AUC = 0.89). Similar conclusions were reached in other studies [104]. Other studies, on the contrary, found that CNN significantly outperformed traditional radiomics feature extraction [90, 94]. Differences among studies may be explained by the different experimental setups, the neural network design, and the size of the training set.

An important aspect to be considered is that the performance of conventional ML approaches saturates quickly with the training set size, as their discriminative abilities are mostly due to the fixed manually engineered features. On the contrary, deep neural networks continue to grow and learn as more training data become available. This phenomenon was quantitatively evaluated by Truhn and colleagues by halving the amount of training data available: the performance of radiomics with respect to the full-size cohort was fairly stable (0.80 vs. 0.81), whereas the AUC of the CNN improved significantly from 0.83 to 0.88 [94]. This implies that DL is the most promising development perspective for lesion classification, as CNN performance is poised to substantially increase as more training data become available.

### 4.4. Deep Learning and Radiomics: Discovering Breast MRI Biomarkers through Deep Learning

“Radiomics” was first mentioned by Gillies et al. in 2010 to describe the high-throughput extraction of quantitative features from images that result in their conversion into mineable data, as well as the process of building predictive models from these data [6]. The success of this approach and terminology was large, to the point that conventional feature extraction methods (including shape, intensity, and texture) are now generally referred to as “radiomic” features.

The process of radiomics generally consists of several closely related steps as follows:

1. Acquire high-quality standardized imaging data and reconstruction.
2. Segment the region of interest (ROI) or the volume of interest (VOI) manually, automatically, or with computer-assisted contouring.
3. Extract a large number of features, in the order of the hundreds.
4. Build clinical prediction models (based on feature selection and machine learning).

The field of radiomics partially overlaps with CADx, but the clinical prediction model may target different outcomes than histopathology, including breast cancer molecular subtype classification, response to therapy, or association to genomics or other omics data. Radiomics features or signature may also play an important role in the discovery of imaging biomarkers [105]. The term “biomarker” refers to a characteristic that is measured objectively, as an indicator of normal biological processes, pathological changes, or response to an intervention. Imaging biomarkers may reflect a general cancer hallmark, e.g., proliferation, metabolism, angiogenesis, and apoptosis; specific molecular interactions; or agnostic features.

Evaluating potential biomarkers or radiomic signatures is beyond the scope of this paper. We refer here to the framework for evaluation of Quantitative Imaging Biomarkers (QIB), proposed by the QIBA Technical Performance Working Group in the paper by Raunig and colleagues [106], but the main principles are also applicable to radiomics [107].

The role of DL in radiomics and biomarker discovery is increasing. In hybrid systems, DL can be applied to anatomical imaging and to perform lesion segmentation prior to feature extraction. DL-based segmentation is faster and more accurate than traditional methods. Automatic methods are preferable in terms of reducing inter- and intraoperator variability [107]. In connection with molecular imaging, it offers better results when it comes to the variability in lesion volume parameters associated with lesion segmentation. DL can also be used also for solving CT-less attenuation correction in hybrid PET/MRI [108–110].

At the same time, DL can be applied directly to breast MR images to extract meaningful features that can be used alongside or replace traditional radiomic feature. While DL has been primary used as a method for joint feature extraction and classification, i.e. to classify tumors as benign or malignant, it is not restricted to image classification. DL can be used to build a wide variety of predictive models [96, 111], as well as predictive biomarkers by summarizing many multimodal breast MR images into compact feature vectors [56].

Thus, the output of the DL neural network will not only provide a lesion classification result but also a quantitative value as a summary of high-dimensional images. As a representation learning technique, DL can provide imaging biomarkers. This is also known as “DL-based radiomics” since the resulting hierarchical features of the hidden layers can be employed as radiomics features. The reproducibility of DL-based features has been less investigated; however, they may be less sensitive to changes in image appearance and quality, as they have been designed and pretrained on natural images that exhibit a large variability in illumination and contrast [112].

In [39], a CNN was designed for breast tumor segmentation, while a subsequent radiogenomic analysis showed that the trained image features had a comparable performance for identifying luminal A subtype breast cancer. DL has also been employed for breast cancer molecular subtype classification based on feature maps of the last fully connected layer [36].

**Figure 6:**
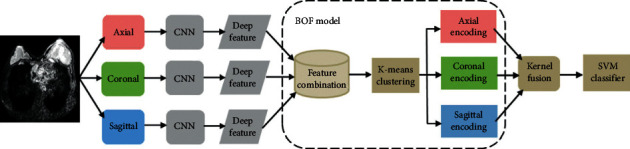
Flow diagram of sentinel lymph node prediction. Reprinted with permission from [113].

In [113], a DWI-based DL model was proposed for the preoperative prediction of sentinel lymph node metastasis in patients with breast cancer. The model combined the CNN and the bag-of-features (BOF) model, which provided relevant feature descriptors based on the DL; accurate feature selection was achieved based on BOF.  Figure 6 describes this model.

In addition, the importance of DL techniques in the evaluation and prediction of neoadjuvant chemotherapy has been described in several papers [33, 38, 114–119]. In [33], a CNN was used for the prediction of pathological complete response to neoadjuvant chemotherapy from baseline breast DCE-MRI. A comparison of different DCE-MRI contrast timepoints with regard to how well their extracted features predicted response to neoadjuvant chemotherapy was performed in [38] within a deep CNN. Extracted features from the precontrast timepoint was determined to be optimal for prediction.

Deep learning methods have been applied to automatically score HER2, a biomarker that determines patients who are eligible for anti-HER2 targeted therapies [120]. This study showed that DL was able to identify cases that are most likely misdiagnosed within the traditional clinical decision-making context.

### 4.5. Specific Characteristics of Breast MRI in Deep Learning Applications

Most DL-based models in computer vision are designed to identify the ground-truth class, assuming that it can be determined with high confidence. In breast MRI, this translates to using pathological information or, less frequently, radiological reports [11, 89] as the ground truth. However, compared to RGB image classification, pathological classes are definitely more ill-defined. First, there is a large interoperator variability among clinicians and pathologists. Secondly, clear-cut discrimination between normal and pathological cases is not always needed or possible [52, 89]. Indeed, medical diagnosis is inherently ambiguous, and DL-based approaches should be able to embrace this by defining a spectrum of lesions and provide fine-grained information to monitor a patient's status and outcome.

The extraction of latent and crucial information is the basis of DL processing. For example, the apparent diffusion coefficient is used as a cancer biomarker in breast MRI in spite of its limitations. The same holds for maximum standardized uptake value in hybrid processing where a single semiquantitative parameter summarizes many high-dimensional image data and represents a predictor for a patient's outcome. DL provides much more information than a conventional imaging parameter and is able to extract the most discriminative key information from multimodal data. The main challenge is how to design a network that can process such high-dimensional dataset in an effective and efficient way. Several examples are provided in Section 4.3 although many current approaches are constrained by the need to leverage pretrained networks on RGB images.

A possible drawback of DL-extracted features is the lack of interpretability [112]. Engineered features are somehow related to characteristics that radiologists use in their clinical assessment, such as lesion size and shape, and may have a direct interpretation. However, this does not necessarily hold true for more complicated features, such as those describing texture. Features extracted from deep neural networks, however, cannot provide a direct mathematical formulation that can explain their behavior. Research is ongoing to accompany DL-based with visual explanations [112].

Another critical aspect is dealing with small medical image datasets. This is tackled by the use of transfer learning coupled with data augmentation, which generates novel training samples by applying random transformations such as rotation, translation, and flipping, thus reducing overfitting [49]. Data augmentation may also help by balancing the often unbalanced classes within medical datasets. Almost the totality of the reviewed literature uses some form of data augmentation although few employ techniques specifically designed for MRI.

An approach that is growing in popularity is the use of generative adversarial networks (GANs) for medical image synthesis [112, 121]. However, synthesizing high-quality 3D images is particularly challenging, and there is the risk to introduce spurious and misleading patterns, e.g., that could mimic lesions in normal cases [122]. This approach has been explored in other pathologies, such as brain MRI [123].

An important aspect of MRI is the wide variability in acquisition parameters across clinical centers. MRI supports wide variations in scanners, acquisition sequences, parameters, and contrast agents. The presence of artifacts and patient motion may further reduce the accuracy of both segmentation and classification [62, 66]. Standardization and repeatability across clinical sites are known issues in all ML applications and radiomic applications in MR [124]. Most studies in literature are single-center studies, which may lead to overestimating the performance over clinical practice. The effect of different acquisition modalities, as well as normalization approaches that can mitigate those differences [51, 68], need to be better explored in the context of deep learning.

## 5. Future Directions and Challenges

The rapid development of AI will lead to a fundamental change in medicine and especially biomedical imaging.

Due to the unique ability of breast MRI to capture both spatial and temporal information, DL needs to be adapted in both architecture and training to fulfill these requirements. Our surveys show that although many applications of DL to breast MRI are emerging, segmentation and lesion classification are today the most mature technologies. However, because images contain rich physiologic, pathologic, and anatomic information, the most important contribution of DL would not be to perform mere lesion classification but to extract latent biological, prognostic, and predictive information. The potential of DL in radiomics is largely untapped as most current approaches are still based on conventional feature extraction [10]. The three main challenges in DL-based biomarkers discovery are excellent prognostic and predictive information, diagnostic uncertainty, and leveraging unlabeled image datasets.

In precision medicine, for example, DL is gaining increasingly relevance for finding biomarkers that predict individual patient outcomes and treatment response. We expect that DL in combination with radiogenomics will provide improved prognostic stratification models. In terms of decision reliability in the clinical settings, DL-based automated systems should identify cases where determining the diagnosis is difficult and requires additional diagnostic tests. DL can be enhanced with Bayesian network modeling, an excellent candidate for uncertainty measurements, to address this challenge. Establishing reproducibility of DL-based features is also a key challenge to overcome for their application in both clinical and research settings.

From the viewpoint of data availability, the biomedical imaging field is a unique position, as raw data are largely available in DICOM format, but annotations are expensive and time-consuming to acquire. Techniques to leverage unlabeled or partially labelled datasets have the potential to greatly advance the application of data hungry DL approaches. Unlabeled datasets can be analyzed based on unsupervised, semisupervised, or self-supervised learning, and the emerging clusters can be used to provide additional information about the subtypes of breast cancer [54].

## 6. The Future of Breast MRI Augmented with AI

The roadmap for the future of AI in breast MRI is to create a safe implementation of AI in which radiologists will not become obsolete as Geoffrey Hinton postulated [125]. On the contrary, the productivity of radiologists will increase based on these intelligent and automated systems. Precision medicine in particular will benefit tremendously from this new technique.

### 6.1. Potential Impact and Implementation Strategy in Breast MRI

The most important task-based categories for the implementation of AI within the scope of breast MRI are as follows:

1. Automated preprocessing such as segmentation, detection, and classification of images: ML techniques are well-established techniques when it comes to automatically detecting breast lesions on mammograms and MRI scans. As a natural next step, DL could be applied to predict the behavior of precancerous lesions and reduce the number of unnecessary and invasive biopsies. Our findings suggests that this is an active and rapidly evolving research area; however, DL-based techniques are mostly still in the technical development phase and require extensive clinical evaluation.
2. Intelligence augmentation: combining AI and the expertise of breast radiologists as a new hybrid intelligence is, in the near future, the most promising direction. Interaction between AI and the human reader needs to be carefully designed and evaluated to maximize accuracy and avoid pitfalls such as under- and overreliance [8]. In our literature review, we found only retrospective, stand-alone performance assessment studies. As the technology becomes more mature, evaluating AI systems in human in the loop scenarios will become of critical importance.
3. Precision medicine and big data: the emergence of radiogenomics which links genomics with imaging phenotypes requires novel AI strategies to process the large amount of data in order to assess breast tumor genetics, behavior, and response to neoadjuvant therapies. The potential of DL-based methods in this context is still largely untapped
4. Decision support systems: AI should be incorporated in decision support systems applied to diagnostic imaging and thus reduce information overload and burnout among breast radiologists.

## 7. Conclusion

Medical decisions in breast cancer patients are made by a detailed interpretation of all relevant patient data including imaging, genomic, and pathologic data. As shown in this article, AI and DL have a major advantage for automatically extracting discriminative features in high-dimensional data over traditional machine learning methods. Thus, AI and DL will impact the breast imaging field tremendously in ways mostly related to quantitative analysis. The multiparametric MRI images provide a plenitude of quantitative information, and thus, various AI and DL techniques will be increasingly applied. Even though there are already automated systems being employed in breast MRI, AI and DL will enhance the importance of multiparametric breast MRI by extracting relevant information from images that will lead to the development of very important biomarkers. Future generations of radiologists will translate breast MRI extracted information to clinical decision-making and will establish important biomarkers for precision medicine.

## Figures and Tables

**Table 1:** Brief overview of common data-driven techniques used in breast MRI.

Technique	Advantages	Disadvantages	References
*Supervised learning*			
Ensemble of decision trees	Decision using branches Variable significance and feature selection are included	Prone to overfitting	[12–14]
			[15, 16]
Random forest	High performance Compared to decision trees	Prone to overfitting	[14, 17, 18]
			[19]
Support vector machines	Transforms nonlinear classification problem into linear one High accuracy	Difficult computation in high-dimensional data space	[20, 21]
			[22, 23]
			[24]
Neural networks	Weights need to be adapted for training Multiclass classification	No strategy to determine network structure	[25–27]
			[28, 29]
			[30, 31]
Deep learning	State-of-the-art in image-derived features	Computationally intensive Hard to interpret	[32, 33]
			[34–36]
			[37–39]

*Unsupervised learning*			
Clustering (*k*-means)	Brief training duration	Number of clusters must be known in advance	[40, 41]
Topological data analysis	Interpretable data mapping Discovery of variable relationships	Divided clusters due to mapping	[28, 42, 43]

**Table 2:** Segmentation applications in breast MRI.

DL technique	Evaluation results	Used dataset	Reference
2D U-net applied slice-by-slice	Dice = 95.90 ± 0.74 Acc = 98.93 ± 0.15 Sn = 95.95 ± 0.69 Sp = 99.34 ± 0.17	42 patients DCE-MRI	[69]
3TP U-net	Dice = 61 ± 11.84 Acc = 99 ± 0.01 Sn = 68.28 ± 9.73 Sp = 100 ± 9.73	35 DCE-MRI 4D data	[60]
GOCS-DLP shape prior based on semantic segmentation based on DL	Dice = 77 ± 13	117 patients DCE-MRI, T2- and T1-weighted images	[70]
2D U-net applied slice-by-slice	Dice = 97	50 DCE-MR images	[71]
Hierarchical multistage U-net with dice loss	Dice = 72 ± 24 Sn = 75 ± 23	Training set: 224 DCE-MRI cases; test set: 48 DCE-MRI cases	[72]
Comparison of 2D U-net and 2D SegNet models with transfer learning from DCE-MRI to DWI	Dice = 72 ± 16	Training: 39 DCE-MR cases and 15 DWI-MR cases; testing: 10 representative DWI-MR slices	[73]
2D U-net applied slice-by-slice to multiplanar sections followed by voxel-level fusion	Dice = 96 ± 0.3 Acc = 99.16 ± 0.13 Sn = 96.85 ± 0.47 Sp = 96.85 ± 0.47	Training: 42 + 88 T1-weighted MRI series (10-fold cross-validation)	[61]

The most common performance measures are the Dice coefficient and the by-voxel accuracy (ACC), sensitivity (Sn), and specificity (Sp). All performance values reported are percentages.

**Table 3:** Detection of breast lesions in breast MRI using DL.

DL technique	Evaluation results	Dataset	References
Model agnostic saliency	TPR = 80 FPs/image = 8	117 subjects DCE-MRI and T1W images	[86]
U-net	Acc = 94.2	67 MR images T1W, T2W, DWI, and DCE-MRI	[87]
Patch-based analysis with ResNet50 backbone	AUC = 0.817	335 MR images of 17 different histological subtypes	[65]
Deep Q-network	Sn = 80 FPs/image = 3.2	117 DCE-MR and T1-weighted images	[63]
Unsupervised saliency analysis and CNN	Acc = 86 ± 2 AUC = 0.94 ± 0.01	193 DCE-MR images	[88]
Two-level U-net and dual-stream CNN	CPM = 64.29	Training: 201 DCE-MR images Testing: 160 DCE-MR images	[64]

**Table 4:** Selected studies reporting classification of breast lesions in breast MRI using DL.

DL technique	Evaluation results	Dataset	Reference
3D CNN from scratch	AUC = 0.739 (2D) AUC = 0.801 (3D)	143 DCE-MR cases (M: 77, B: 66)	[35]
CNN (ResNet50) fine-tuned	AUC = 0.97–0.99	Training: 33 patients with 153 lesions (M: 91, B: 62) Testing: 74 patients with 74 lesions (M: 48, B: 26)	[90]
Cross-modal DL (mammography and MR), trained from scratch	Acc = 94 AUC = 0.98	123 DCE-MR + T1W 282 mammography images	[91]
Dense convolutional LSTM	Acc = 0.847 Precision = 78.2 Sn = 81.5	72 lesions (M: 27, B: 45) DCE-MRI and DWI-MRI	[92]
DenseNet	AUC = 0.811	576 lesions (M: 368, B: 149, FU: 59) Ultrafast DCE-MRI, T2, and DWI	[93]
CNN (AlexNet) fine-tuned from ImageNet on the second postcontrast frame, LSTM model for final prediction	Acc = 76 AUC = 0.76	42 DCE-MR images, 67 lesions (M: 42, B: 25) 10-fold cross-validation	[59]
CNN (ResNet34) fine-tuned best three inputs out of 85 combinations	AUC = 0.88 (95% confidence interval: 0.86–0.89)	447 patients, 1294 lesions (M: 787, B: 507) multiparametric DCE-MR + T2W 10-fold cross-validation	[94]
MIP + off-the-shelf CNN (VGG) + SVM	AUC = 0.88 ± 0.01	690 DCE-MR cases (M: 478, B: 212) 5-fold cross-validation	[32]
Multiscale 3D CNN (trained from scratch) inputs: five timepoints T1W DCE-MR and T2W	AUC = 0.89 ± 0.01	408 patients (M: 305, B: 103) multiparametric DCE-MR 5-fold cross-validation	[95]
Off-the-shelf CNN (VGG) + SVM target: different molecular subtypes	AUC = 0.65 (pretrained) AUC = 0.58 (from scratch)	270 DCE-MR images (90 luminal A, 180 other 3 subtypes) 10-fold cross-validation	[96]
3TP-CNN pretrained on ImageNet	Acc = 74 AUC = 0.81 F1 = 0.78	39 lesions (M: 36, B: 22) DCE-MRI sequences 10-fold cross-validation	[97]
Three-channel (pre- and postcontrast) CNN (VGG) fine-tuned for classification	AUC = 0.88 (CNN + LSTM) AUC = 0.84 (CNN-only)	703 DCE-MR dataset (M: 482, B: 221) 80% training + validation, 20% testing	[58]
3D ResNet trained from scratch with ad hoc embedding loss weakly supervised localization with feature correlation attention map	Acc = 85.5 AUC = 0.902	1715 subjects (M: 1137, B: 578) Training: 1204 subjects Testing: 346 subjects	[98]

For each study, we report the number of histologically verified benign (*B*) and malignant (*M*) lesions or cases; benign lesions without biopsy with at least 12-month follow-up (FU) are also indicated. Histology is used as ground truth in all studies.
